# Upregulation of HSPA1A/HSPA1B/HSPA7 and Downregulation of HSPA9 Were Related to Poor Survival in Colon Cancer

**DOI:** 10.3389/fonc.2021.749673

**Published:** 2021-10-26

**Authors:** Yufeng Guan, Xianjun Zhu, Junjie Liang, Min Wei, Shan Huang, Xiaofen Pan

**Affiliations:** ^1^ Department of General Surgery, Guangzhou Panyu Central Hospital, Guangzhou, China; ^2^ Department of Oncology, The Seventh Affiliated Hospital, Sun Yat-sen University, Shenzhen, China

**Keywords:** colon cancer, HSP70, HSPA, HSPA1A, HSPA1B, HSPA7, HSPA9, survival

## Abstract

The human HSP70 family is a type of heat shock protein (HSP), consisting of 13 members encoded by the HSPA genes. HSPs play important roles in regulating cellular responses and functions during carcinogenesis, but their relationship with colon cancer is unclear. In our study, we found that the expressions of HSPA1B, HSPA4, HSPA5, HSPA6, HSPA8, HSPA9, HSPA13, and HSPA14 were significantly increased, while those of HSPA1A, HSPA2, HSPA7, and HSPA12B were significantly decreased in colon cancer tissues. The expression of HSPA gene family members was associated with some clinicopathological characteristics, including age, gender, TNM stage, pathological stage, and CEA level. Furthermore, the Kaplan–Meier method and Cox regression analysis showed that high HSPA1A, HSPA1B, and HSPA7 expressions were related to unfavorable survival, and high HSPA9 was associated with favorable survival. The relationships between HSPA1A and HSPA9 expression and survival were validated in the GEO dataset, and the HSPA1A and HSPA9 protein expression differences between colon cancer tissues and normal tissues were validated in the UALCAN database. Methylation of HSPA1A and HSPA9 was also analyzed, and it was found that the methylation of the HSPA1A promoter was significantly increased, and the methylation of the HSPA9 promoter was significantly decreased in colon cancer tissues. Increasing the methylation level of the HSPA1A gene and decreasing the methylation level of HSPA9 were related to favorable prognosis. The expression difference of HSPA1A/HSPA1B/HSPA7/HSPA9 was verified in colon cancer cell lines and colonic epithelial cells. Gene ontology analysis was used to screen signal pathways related to HSPA1A-, HSPA1B-, HSPA7-, and HSPA9- high phenotype. In summary, the increased expressions of HSPA1A1, HSPA1B, and HSPA7 were associated with poor prognosis, while that of HSPA9 was related to favorable prognosis for colon cancer patients.

## Introduction

Colon cancer is one of the most common malignant tumors in the world. There were about 18.1 million new cancer cases and 9.6 million cancer-related deaths in 2018. Among all types of cancers, colon cancer ranks third in terms of incidence (1,096,601 new cases) and fourth in mortality (551,269 deaths) (1). The standard treatment of early-stage colon cancer is surgical resection combination with adjuvant therapy if appropriate. However, about 30% of the patients develop local recurrence or distant metastasis after curative therapy (2). For these patients, system therapy is the recommended therapy. Although the treatment efficacy has been improved in the past few years, the prognosis of patients with advanced or recurrent colon cancer is still poor, with an overall survival interval of 13–17 months (3). Therefore, it is an urgent need to discover new biomarkers with prognostic and therapeutic value.

Heat shock proteins (HSPs) are a group of proteins that function to reverse or inhibit denaturation or unfolding of cellular proteins in response to stress or high temperature (4, 5). The types of heat shock proteins include HSP27, HSP40, HSP60, HSP70, HSP90, and large HSPs (HSP110 and glucoseregulated protein 170, GRP170) (6). The human HSP70 family consists of 13 members encoded by the HSPA genes, including HSPA1A, HSPA1B, HSPA2, HSPA4, HSPA5, HSPA6, HSPA7, HSPA8, HSPA9, HSPA12A, HSPA12B, HSPA13, and HSPA14 (7). HSP70 proteins have highly conserved domain structures, including a 44-kDa N-terminal ATPase domain, an 18-kDa substrate-binding domain, and a 10-kDa C-terminal domain (4). It has been reported that HSP70 was increased in colorectal cancer tissues (8). But there are few reports about the expression and clinical significance of distinct HSP70 family members in colon cancer. Therefore, in the current study, we investigated the expression of distinct HSPA family members in colon cancer using the public database. Moreover, the relationships between HSPA family members’ expression and clinical pathological parameters and survival were assessed. We found that increased expressions of HSPA1A1, HSPA1B, and HSPA7 were associated with poor prognosis, while that of HSPA9 was related to favorable prognosis for colon cancer patients. The results were further verified in independent datasets. Furthermore, GO ontology analysis was performed to screen the signaling pathways related to HSPA1A, HSPA1B, HSPA7, and HSPA9 in colon cancer.

## Method

### Datasets and Clinical Information

Gene expression data and corresponding clinical information were downloaded from the TCGA database (project ID: TCGA-COAD) (https://portal.gdc.cancer.gov/). In total, information on 480 cases of colon cancer tissues and 41 adjacent normal tissues was downloaded. Clinical information included age, gender, TNM stage, pathologic stage, CEA level, and survival time. Clinicopathologic characteristics are shown in **Table S1**. The expression level of members of the HSPA family and the correlation to clinicopathologic characteristics and survival were analyzed. The Clinical Proteomic Tumor Analysis Consortium (CPTAC) (9) database (http://ualcan.path.uab.edu/analysis-prot.html) was used to verify the protein expression level of HSPA1A and HSPA9. Z-values represent standard deviations from the median across samples for the given cancer type. Log^2^ Spectral count ratio values from CPTAC were firstly normalized within each sample profile, then normalized across samples. Correlation of HSPA1A and HSPA9 expression with survival was verified in the GSE28122 dataset from the GEO database (www.ncbi.nlm.nih.gov/geo/). The UALCAN (10) database (http://ualcan.path.uab.edu/index.html) was used to compare the DNA methylation level of HSPA1A and HSPA9 in normal tissues and colon cancer tissues. The MethSurv (11) database (https://biit.cs.ut.ee/methsurv/) was used to analyze the correlation of the DNA methylation level of HSPA1A and HSPA9 with survival in colon cancer patients.

### Gene Ontology Analysis

Analysis was conducted by R software (R x64 3.6.3). Patients were classified into HSPA1A/HSPA1B/HSPA7/HSPA9 high and low groups using the median values as cutoff. Gene expression difference between high and low groups was analyzed (12). Genes with P<0.05 were considered as differential genes. Differential genes then were used to conduct enrichment analysis by the clusterProfiler package (13). Histograms and network charts were used to visualize the enriched pathways.

### RNA Extraction and Quantitative Real-Time Polymerase Chain Reaction (qRT-PCR) Analysis of Colon Cancer Cells

Colon cancer cell lines HCT116 and HT29 and normal colonic epithelial cell line CP-H040 were purchased from Procell Company (Wuhan, China). Total RNA of the cell lines was extracted using the TRIzol reagent (Invitrogen, Carlsbad, USA). cDNA was obtained by reverse transcription according to the Takara RT-PCR kit (Takara, Japan) manufacturer’s instructions. Primer sequences were shown as follows: (1) HSPA1A up, 5′-GCC TTT CCA AGA TTG CTG TT-3′; HSPA1A dn, 5′- TCA ACA TTG CAA ACA CAG GA-3′; (2) HSPA1B up, 5′-AGG GTG TTT CGT TCC CTT TA- 3′; HSPA1B dn, 5′-CAT TCC CAG CCT TTG TAG TG-3′; (3) HSPA7 up, 5′-CAA CCT GCT GGG GCG TTT TGA-3′; HSPA7 dn, 5′-CCG GCC CTT GTC ATT GGT GAT CTT-3′; (4) HSPA9 up, 5′- TGG AAT GCC GGC CAA GCG AC-3′; HSPA9 dn, 5′-GCC TCA ACC CAG GCA TCA CCA-3′; GAPDH up, 5′-GAA ATC CCA TCA CCA TCT TCC AGG-3′; GAPDH dn, 5′-GAG CCC CAGCCT TCT CCA TG-3′. qRT-PCR was performed according to the manufacturer’s instructions of the SYBR Green reagent (ABI, USA).

### Statistical Analyses

Statistical analyses were conducted by R software (R x64 3.6.3). The unpaired comparisons of HSPA family members expression between normal and tumor samples were analyzed by Wilcoxon rank-sum test, and the paired comparisons were analyzed by Wilcoxon signed-rank test. The HSPA family members’ expression differences in patients with different clinical parameters were analyzed by Wilcoxon signed-rank test. Logistic regression was conducted to analyze the correlation of HSPA family members with clinicopathological characteristics in colon cancer. The Kaplan–Meier method was performed to analyze the relationship between different expression levels of HSPA family members and survival of colon cancer patients. The correlation of HSPA1A/HSPA1B/HSPA7/HSPA9 and clinicopathologic parameters with survival was estimated by univariate and multivariate analyses.

## Results

### Differential Expression of HSPA Family Members in Colon Cancer and Normal Tissues

**Figure 1:**
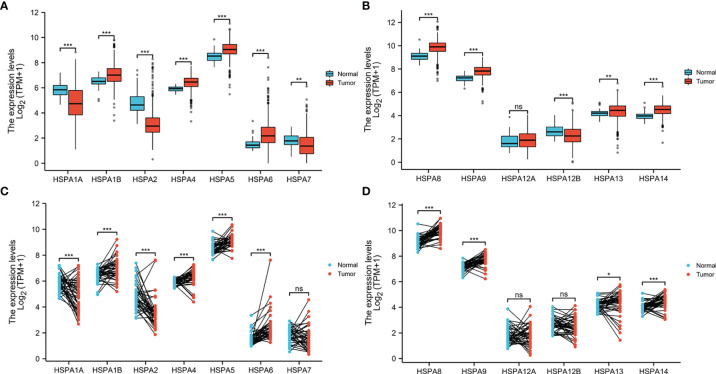
Differential expression of HSPA family members in colon cancer and normal tissues. **(A)** HSPA1A, HSPA1B, HSPA2, HSPA4, HSPA5, HSPA6, and HSPA7 expression in colon cancer and normal tissues by unpaired comparison. **(B)** HSPA8, HSPA9, HSPA12A, HSPA12B, HSPA13, and HSPA14 expression in colon cancer and normal tissues by unpaired comparison. **(C)** HSPA1A, HSPA1B, HSPA2, HSPA4, HSPA5, HSPA6, and HSPA7 expression in colon cancer and normal tissues by paired comparison. **(D)** HSPA8, HSPA9, HSPA12A, HSPA12B, HSPA13, and HSPA14 expression in colon cancer and normal tissues by paired comparison (Wilcoxon rank-sum test for unpaired comparisons and Wilcoxon signed-rank test for paired comparisons. ns, non significant; *P < 0.05; **P < 0.01; ***P < 0.001).

Wilcoxon rank-sum test (for unpaired comparisons) and Wilcoxon signed-rank test (for paired comparisons) were used to compare the HSPA family members’ expression level between 480 colon cancer tissues and 41 adjacent nontumor tissues. Unpaired comparisons showed that HSPA1B (*P*<0.001), HSPA4 (*P*<0.001), HSPA5 (*P*<0.001), HSPA6 (*P*<0.001), HSPA8 (*P*<0.001), HSPA9 (*P*<0.001), HSPA13 (*P*<0.001), and HSPA14 (*P*<0.001) were upregulated in colon cancer and HSPA1A (*P*<0.001), HSPA2 (*P*<0.001), HSPA7 (*P*<0.01), and HSPA12B (*P*<0.001) were downregulated. HSPA12A expression showed no significant difference between colon cancer tissues and normal tissues (**Figures 1A, B**). Paired comparisons showed consistent results except that no difference was observed in HSPA7 and HSPA12B (**Figures 1C, D**).

### Correlation of HSPA Family Expression With Clinical Pathological Characteristics in Colon Cancer

**Figure 2:**
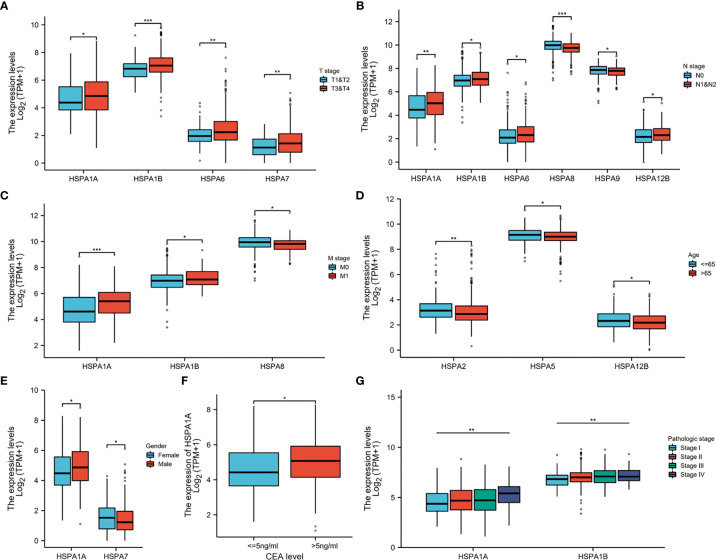
Correlation of HSPA family expression with clinical pathological characteristics in colon cancer. **(A)** Correlation of HSPA1A, HSPA1B, HSPA6, and HSPA7 expression with T stage. **(B)** Correlation of HSPA1A, HSPA1B, HSPA6, HSPA8, HSPA9, and HSPA12B expression with N stage. **(C)** Correlation of HSPA1A, HSPA1B, and HSPA8 expression with M stage. **(D)** Correlation of HSPA2, HSPA5, and HSPA12B expression with age. **(E)** Correlation of HSPA1A and HSPA7 expression with gender. **(F)** Correlation of HSPA1A expression with CEA level (Wilcoxon rank-sum test. *P < 0.05; **P < 0.01; ***P < 0.001). **(G)** Correlation of HSPA1A and HSPA1B with pathologic stage (Kruskal–Wallis test. **P < 0.01).

Wilcoxon rank-sum test was used to compare the HSPA family expression in patients with different clinicopathologic parameters. Results showed that patients with T3-4 stage disease had higher expression of HSPA1A, HSPA1B, HSPA6, and HSPA7 than patients with T1-2 disease (**Figure 2A**). Patients with lymph node invasion had increased expression of HSPA1A, HSPA1B, HSPA6, and HSPA12B and decreased expression of HSPA8 and HSPA9 (**Figure 2B**). HSPA1A and HSPA1B were upregulated, while HSPA8 was downregulated in patients with metastatic disease (**Figure 2C**). HSPA2, HSPA5, and HSPA12B expressions were higher in patients younger than 65 years (**Figure 2D**). Male patients had higher expression of HSPA1A, and female patients had higher expression of HSPA7 (**Figure 2E**). Patients with an elevated CEA level had higher HSPA1A expression (**Figure 2F**). Kruskal–Wallis test was performed to analyze the correlation of the HSPA family with the pathologic stage. Results showed that the expression level of HSPA1A and HSPA1B increased as the pathologic stage increased (**Figure 2G**). The results of the correlation of other HSPA family members’ expression with clinical pathological characteristics are shown in **Figures S1**, **S2**.

Logistic regression analysis with HSPA family members as dependent variables showed that HSPA1A expression was associated with T stage (OR=1.719 for T3-4 *vs.*T1-2, 95%CI, 1.090-2.740, *P*=0.021), N stage (OR=1.688 for N1-2 *vs.*N0, 95%CI, 1.170-2.446, *P*=0.005), M stage (OR=2.855 for M1 *vs.*M0, 95%CI, 1.634-5.168, *P*<0.001), and CEA level (OR=1.870 for CEA>5ng/ml *vs.*CEA<5ng/ml, 95%CI, 1.163-3.027, *P*=0.010). HSPA1B expression was associated with T stage (OR=1.817 for T3-T4 *vs.*T1-T2, 95%CI, 1.150-2.903, *P*=0.011), while HSPA2 expression was associated with age (OR=0.592 for >65y *vs.*<=65y, 95%CI, 0.409-0.855, *P*=0.005). Elevated HSPA4 expression was associated with N stage (OR=0.636 for N1-2 *vs.*N0, 95%CI, 0.439-0.917, *P*=0.016). HSPA5 was related to age (OR=0.682 for >65y *vs.*<=65y, 95%CI, 0.472-0.983, *P*=0.041), T stage (OR=1.719 for T3-T4 *vs.*T1-T2, 95%CI, 1.090-2.740, *P*=0.021), and N stage (OR=1.467 for N1-2 *vs.*N0, 95%CI, 1.017-2.120, *P*=0.041). The HSPA6 expression level was associated with T stage (OR=1.871 for T3-T4 *vs.*T1-T2, 95%CI, 1.150-2.903, *P*=0.011) and N stage (OR=1.467 for N1-2 *vs.*N0, 95%CI, 1.017-2.120, *P*=0.041). HSPA7 expression was associated with gender (OR=0.603 for male *vs.*female, 95%CI, 0.419-0.865, *P*=0.006). Upregulation of HSPA8 was correlated with age (OR=1.467 for >65y *vs.*<=65y, 95%CI, 1.017-2.120, *P*=0.041), N stage (OR=0.478 for N1-2 *vs.*N0, 95%CI, 0.329-0.692, *P*<0.001), and M stage (OR=0.528 for M1 *vs.*M0, 95%CI, 0.305-0.901, *P*=0.021). The expression of HSPA9, HSPA12A, HSPA12B, HSPA13, and HSPA14 showed no association to age, gender, TNM stage, pathologic stage, and CEA level (**Table 1**).

**Table 1:** Correlation of HSPA family expression with clinical pathological characteristics in colon cancer analyzed by Logistic regression.

	Characteristics	Total (N)	Odds Ratio (OR)	P value
HSPA1A	Age (>65 *vs.*<=65)	478	0.901 (0.625-1.298)	0.576
	Gender (Male *vs.*Female)	478	1.400 (0.977-2.010)	0.067
	T stage (T3&T4 *vs.*T1&T2)	477	1.719 (1.090-2.740)	**0.021^†^**
	N stage (N1&N2 *vs.*N0)	478	1.688 (1.170-2.446)	**0.005^†^**
	M stage (M1 *vs.*M0)	415	2.855 (1.634-5.168)	**<0.001^†^**
	CEA level (>5ng/ml *vs.*<=5ng/ml)	303	1.870 (1.163-3.027)	**0.01^†^**
HSPA1B	Age (>65 *vs.*<=65)	478	0.901 (0.625-1.298)	0.576
	Gender (Male *vs.*Female)	478	1.265 (0.883-1.815)	0.2
	T stage (T3&T4 *vs.*T1&T2)	477	1.817 (1.150-2.903)	**0.011^†^**
	N stage (N1&N2 *vs.*N0)	478	1.368 (0.949-1.975)	0.094
	M stage (M1 *vs.*M0)	415	1.230 (0.727-2.092)	0.441
	CEA level (>5 *vs.*<=5)	303	0.741 (0.459-1.189)	0.216
HSPA2	Age (>65 *vs.*<=65)	478	0.592 (0.409-0.855)	**0.005^†^**
	Gender (Male *vs.*Female)	478	1.223 (0.854-1.754)	0.272
	T stage (T3&T4 *vs.*T1&T2)	477	1.311 (0.835-2.071)	0.241
	N stage (N1&N2 *vs.*N0)	478	1.190 (0.826-1.716)	0.352
	M stage (M1 *vs.*M0)	415	1.313 (0.775-2.243)	0.313
	CEA level (>5 *vs.*<=5)	303	1.357 (0.846-2.185)	0.206
HSPA4	Age (>65 *vs.*<=65)	478	0.933 (0.647-1.344)	0.709
	Gender (Male *vs.*Female)	478	0.764 (0.533-1.095)	0.143
	T stage (T3&T4 *vs.*T1&T2)	477	1.311 (0.835-2.071)	0.241
	N stage (N1&N2 *vs.*N0)	478	0.636 (0.439-0.917)	**0.016^†^**
	M stage (M1 *vs.*M0)	415	0.753 (0.441-1.276)	0.294
	CEA level (>5 *vs.*<=5)	303	0.631 (0.390-1.013)	0.058
HSPA5	Age (>65 *vs.*<=65)	478	0.682 (0.472-0.983)	**0.041^†^**
	Gender (Male *vs.*Female)	478	0.764 (0.533-1.095)	0.143
	T stage (T3&T4 *vs.*T1&T2)	477	1.719 (1.090-2.740)	**0.021^†^**
	N stage (N1&N2 *vs.*N0)	478	1.467 (1.017-2.120)	**0.041^†^**
	M stage (M1 *vs.*M0)	415	0.898 (0.527-1.521)	0.689
	CEA level (>5 *vs.*<=5)	303	0.942 (0.588-1.510)	0.804
HSPA6	Age (>65 *vs.*<=65)	478	1.035 (0.718-1.492)	0.852
	Gender (Male *vs.*Female)	478	1.000 (0.698-1.432)	1.000
	T stage (T3&T4 *vs.*T1&T2)	477	1.817 (1.150-2.903)	**0.011^†^**
	N stage (N1&N2 *vs.*N0)	478	1.467 (1.017-2.120)	**0.041^†^**
	M stage (M1 *vs.*M0)	415	1.556 (0.917-2.676)	0.104
	CEA level (>5 *vs.*<=5)	303	1.280 (0.799-2.059)	0.306
HSPA7	Age (>65 *vs.*<=65)	478	1.035 (0.718-1.492)	0.852
	Gender (Male *vs.*Female)	478	0.603 (0.419-0.865)	**0.006^†^**
	T stage (T3&T4 *vs.*T1&T2)	477	1.311 (0.835-2.071)	0.241
	N stage (N1&N2 *vs.*N0)	478	1.072 (0.744-1.545)	0.709
	M stage (M1 *vs.*M0)	415	0.936 (0.551-1.585)	0.805
	CEA level (>5 *vs.*<=5)	303	1.091 (0.680-1.753)	0.718
HSPA8	Age (>65 *vs.*<=65)	478	1.467 (1.017-2.120)	**0.041^†^**
	Gender (Male *vs.*Female)	478	0.874 (0.610-1.252)	0.464
	T stage (T3&T4 *vs.*T1&T2)	477	0.857 (0.544-1.347)	0.504
	N stage (N1&N2 *vs.*N0)	478	0.478 (0.329-0.692)	**<0.001^†^**
	M stage (M1 *vs.*M0)	415	0.528 (0.305-0.901)	**0.021^†^**
	CEA level (>5 *vs.*<=5)	303	0.654 (0.405-1.049)	0.079
HSPA9	Age (>65 *vs.*<=65)	478	0.966 (0.670-1.392)	0.852
	Gender (Male *vs.*Female)	478	0.790 (0.551-1.132)	0.200
	T stage (T3&T4 *vs.*T1&T2)	477	1.179 (0.750-1.857)	0.476
	N stage (N1&N2 *vs.*N0)	478	0.757 (0.524-1.091)	0.136
	M stage (M1 *vs.*M0)	415	0.665 (0.387-1.129)	0.134
	CEA level (>5 *vs.*<=5)	303	0.657 (0.407-1.055)	0.084
HSPA12A	Age (>65 *vs.*<=65)	478	0.812 (0.563-1.170)	0.264
	Gender (Male *vs.*Female)	478	0.874 (0.610-1.252)	0.464
	T stage (T3&T4 *vs.*T1&T2)	477	1.298 (0.826-2.049)	0.26
	N stage (N1&N2 *vs.*N0)	478	1.072 (0.744-1.545)	0.709
	M stage (M1 *vs.*M0)	415	1.592 (0.938-2.738)	0.088
	CEA level (>5 *vs.*<=5)	303	1.530 (0.954-2.468)	0.079
HSPA12B	Age (>65 *vs.*<=65)	478	0.784 (0.543-1.130)	0.193
	Gender (Male *vs.*Female)	478	0.874 (0.610-1.252)	0.464
	T stage (T3&T4 *vs.*T1&T2)	477	1.311 (0.835-2.071)	0.241
	N stage (N1&N2 *vs.*N0)	478	1.321 (0.916-1.907)	0.136
	M stage (M1 *vs.*M0)	415	0.727 (0.426-1.233)	0.239
	CEA level (>5 *vs.*<=5)	303	0.862 (0.537-1.383)	0.537
HSPA13	Age (>65 *vs.*<=65)	478	0.933 (0.647-1.344)	0.709
	Gender (Male *vs.*Female)	478	1.309 (0.914-1.877)	0.143
	T stage (T3&T4 *vs.*T1&T2)	477	1.243 (0.791-1.960)	0.346
	N stage (N1&N2 *vs.*N0)	478	0.966 (0.670-1.392)	0.852
	M stage (M1 *vs.*M0)	415	0.628 (0.365-1.066)	0.088
	CEA level (>5 *vs.*<=5)	303	1.058 (0.660-1.696)	0.816
HSPA14	Age (>65 *vs.*<=65)	478	0.812 (0.563-1.170)	0.264
	Gender (Male *vs.*Female)	478	0.967 (0.675-1.385)	0.855
	T stage (T3&T4 *vs.*T1&T2)	477	0.771 (0.488-1.211)	0.26
	N stage (N1&N2 *vs.*N0)	478	0.757 (0.524-1.091)	0.136
	M stage (M1 *vs.*M0)	415	0.782 (0.459-1.325)	0.362
	CEA level (>5 *vs.*<=5)	303	1.080 (0.674-1.731)	0.75

^†^Bold values represent p < 0.05.

### Survivals of Patients With Different HSPA Family Member Expression Levels Were Estimated by Kaplan–Meier Method

**Figure 3:**
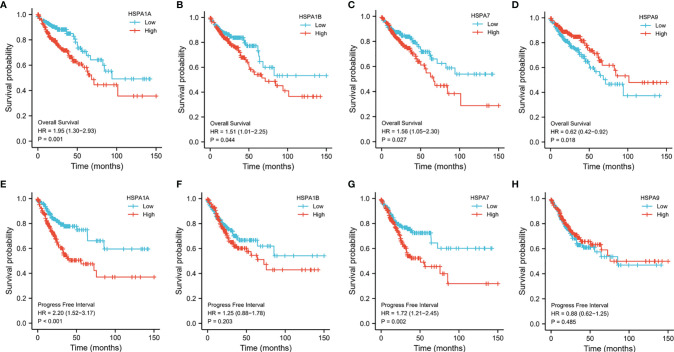
Survivals analysis of patients with different HSPA1A, HSP1B, HSPA7, and HSPA9 expression levels by Kaplan–Meier method. **(A)** Overall survival for HSPA1A. **(B)** Overall survival for HSPA1B. **(C)** Overall survival for HSPA7. **(D)** Overall survival for HSPA9. **(E)** Progress-free interval for HSPA1A. **(F)** Progress free interval for HSPA1B. **(G)** Progress free interval for HSPA7. **(H)** Progress free interval for HSPA9.

The Kaplan–Meier method was used to estimate the survival of colon cancer patients with different expression levels of HSPA family members. Results showed that patients with a high expression level of HSPA1A, HSPA1B, and HSPA7 showed worse overall survival (for HSPA1A, HR=1.95, 95%CI, 1.30-2.93, *P*=0.001; for HSPA1B, HR=1.51, 95%CI, 1.01-2.25, *P*=0.044; for HSPA7, HR=1.56, 95%CI, 1.05-2.30, *P*=0.027) (**Figures 3A**–**C**). However, upregulation of HSPA9 was associated with better overall survival (HR 0.62, 95%CI, 0.42-0.92, *P*=0.018) (**Figure 3D**). Increased HSPA1A and HSPA7 were also related to poor progression-free interval (for HSPA1A, HR=2.20, 95%CI, 1.52-3.17, *P*<0.001; for HSPA7, HR=1.72, 95%CI, 1.21-2.45, *P*=0.002) (**Figures 3E, G**). Howerver, expression level of HSPA1B and HSPA9 was not related to progression-free interval (**Figures 3F, H**). Survival of patients with different expression levels of other HSPA family members is shown in **Figure S3**.

### Prognostic Value of HSPA1A, HSPA1B, HSPA7, and HSPA9 in Colon Cancer

Univariate regression analysis was performed to explore the correlation of HSPA1A, HSPA1B, HSPA7, and HSPA9 expression with survival in colon cancer patients. Results showed that high expression of HSPA1A (HR=1.950, 95%CI, 1.300-2.926, *P*=0.001), HSPA1B (HR=1.508, 95%CI, 1.012-2.248, *P*=0.044), HSPA7 (HR=1.557, 95%CI, 1.053-2.302, *P*=0.027), HSPA9 (HR=0.620, 95%CI, 0.418-0.920, *P*=0.018), and other clinical parameters were associated with survival, such as T stage (for T3-4 *vs* T1-2, HR=2.560, 95%CI, 1.175-5.578, *P*=0.018), N stage (for N1-2 *vs* N0, HR=2.592, 95%CI, 1.743-3.855, *P*<0.001), M stage (for M1 *vs* M0, HR=4.193, 95%CI, 2.683-6.554, *P*<0.001), and CEA level (for >5 ng/ml *vs* <5 ng/ml, HR=3.128, 95%CI, 1.788-5.471, *P*<0.001). Multivariate Cox regression was further used to estimate whether the prognostic values of HSPA1A, HSPA1B, HSPA7, and HSPA9 were independent. It was shown that HSPA1B (HR=2.745, 95%CI, 1.188-6.339, *P*=0.018) was an independent factor for survival in colon cancer (**Table 2**).

**Table 2:** Univariate analysis and multivariate cox regression analysis.

Characteristics	Total (N)	Univariate analysis		Multivariate analysis	
		Hazard ratio (95% CI)	P value	Hazard ratio (95% CI)	P value
Age (>65 *vs.*<=65)	477	1.610 (1.052-2.463)	**0.028**	3.355 (1.360-8.276)	**0.009^†^**
Gender (Male *vs.*Female)	477	1.101 (0.746-1.625)	0.627		
T stage (T3 *vs.*T1&T2)	416	2.560 (1.175-5.578)	**0.018**	1.534 (0.328-7.178)	0.587
N stage (N1&N2 *vs.*N0)	477	2.592 (1.743-3.855)	**<0.001**	3.708 (1.459-9.425)	**0.006^†^**
M stage (M1 *vs.*M0)	414	4.193 (2.683-6.554)	**<0.001**	0.802 (0.289-2.223)	0.671
CEA level (>5 *vs.*<=5)	302	3.128 (1.788-5.471)	**<0.001**	2.199 (0.962-5.028)	0.062
HSPA1A (High *vs.*Low)	477	1.950 (1.300-2.926)	**0.001**	0.969 (0.447-2.101)	0.937
HSPA1B (High *vs.*Low)	477	1.508 (1.012-2.248)	**0.044**	2.745 (1.188-6.339)	**0.018^†^**
HSPA7 (High *vs.*Low)	477	1.557 (1.053-2.302)	**0.027**	1.569 (0.735-3.350)	0.244
HSPA9 (High *vs.*Low)	477	0.620 (0.418-0.920)	**0.018**	0.883 (0.417-1.870)	0.745

^†^Bold values represent p < 0.05.

### Verification of HSPA1A and HSPA9 Protein Expression and the Correlation to Survival in Colon Cancer

**Figure 4:**
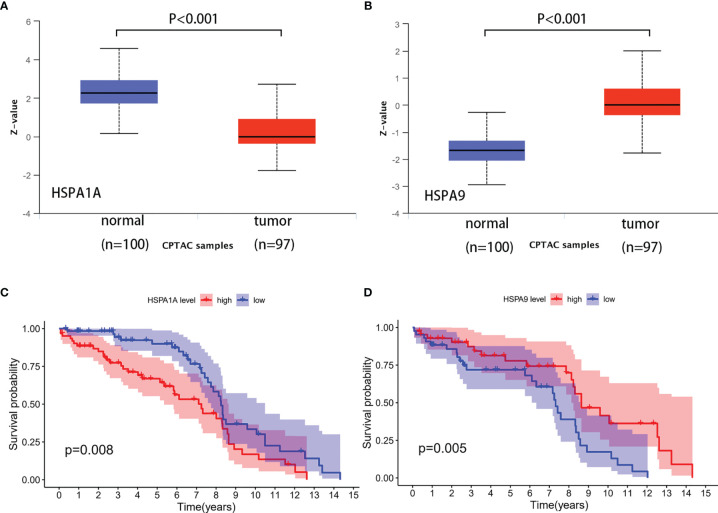
Verification of HSPA1A and HSPA9 protein expression and the correlation to survival in colon cancer. **(A)** HSPA1A protein expression. **(B)** HSPA9 protein expression. **(C)** Survival of patients with high and low HSPA1A expression (sample from GSE28122). **(D)** Survival of patients with high and low HSPA9 expression (sample from GSE28122).

HSPA1A and HSPA9 protein expressions in normal tissues and colon cancer tissues were analyzed by the Clinical Proteomic Tumor Analysis Consortium (CPTAC) (9) database (http://ualcan.path.uab.edu/analysis-prot.html). A total of 100 normal tissues and 97 colon cancer tissues were included. Results showed that HSPA1A protein was decreased (*P*<0.001) and HSPA9 was increased (*P*<0.001) in colon cancer tissues (**Figures 4A, B**). The result was consistent with the change of HSPA1A and HSPA9 mRNA in the TCGA database. Information of the GSE28122 dataset was downloaded from the GEO database (www.ncbi.nlm.nih.gov/geo/). Survival of patients with different HSPA1A and HSPA9 expression levels was compared. It was revealed that patients with high HSPA1A and low HSPA9 showed poor prognosis (**Figures 4C, D**), which was consistent with the results from the TCGA database.

### Analysis of DNA Methylation of HSPA1A and HSPA9 Genes in Normal and Colon Cancer Tissues

**Figure 5:**
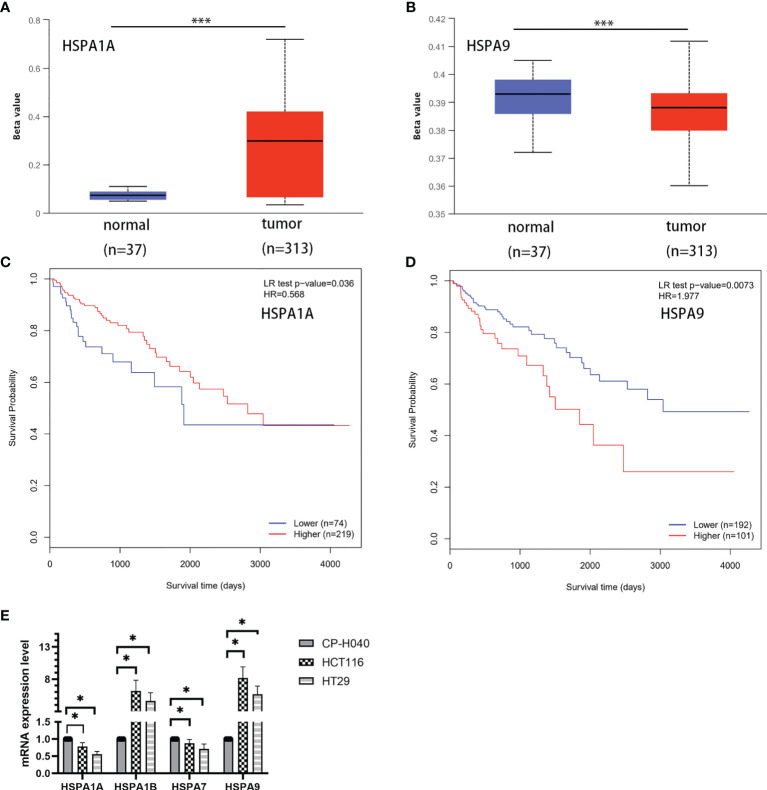
Analysis of DNA methylation of HSPA1A and HSPA9 genes in normal and colon cancer tissues and the correlation with survival. **(A)** Methylation of HSPA1A promoter in colon cancer tissues and normal tissues. **(B)** Methylation of HSPA9 promoter in colon cancer tissues and normal tissues (***P < 0.001). **(C)** Survival of patients with high and low HSPA1A promoter methylation level. **(D)** Survival of patients with high and low HSPA9 promoter methylation level. **(E)** Expression of HSPA1A/HSPA1B/HSPA7/HSPA9 mRNA in colon cancer cells (HCT116 and HT29) and colonic epithelial cells (CP-H040) (data were presented as mean ± SD; *P < 0.05).

The UALCAN database (http://ualcan.path.uab.edu/index.html) was used to compare the DNA methylation of HSPA1A and HSPA9 genes in normal tissues and colon cancer tissues. Results showed that promoter methylation of the HSPA1A gene in colon cancer tissues was significantly higher than normal tissues (*P*<0.001) (**Figure 5A**), and methylation of HSPA9 was significantly lower in colon cancer tissues (*P*<0.001) (**Figure 5B**). The MethSurv (11) database (https://biit.cs.ut.ee/methsurv/) was used to analyze the correlation of DNA methylation of HSPA1A and HSPA9 with survival. It was revealed that patients with high HSPA1A promoter methylation had better survival (HR=0.568, 95% CI, 0.342-0.944, *P*=0.036) (**Figure 5C**), while high HSPA9 promoter methylation was related to poor prognosis (HR=1.977, 95% CI, 1.216-3.213, *P*=0.0073) (**Figure 5D**).

### Verification of HSPA1A/HSPA1B/HSPA7/HSPA9 mRNA Expression in Colon Cancer Cell Lines

To verify the expression difference of HSPA1A/HSA1B/HSPA7/HSPA9 in the TCGA database, we compared the expression of these genes in colon cancer cells (HCT116 and HT29) and colonic epithelial cells (CP-H040). Results showed that the expressions of HSPA1A and HSPA7 were downregulated in colon cancer cell lines. HSAP1B and HSPA9 were upregulated in colon cancer cell lines (**Figure 5E**).

### HSPA1A-, HSPA1B-, HSPA7-, and HSPA9-Related Signaling Pathways Identified by Gene Ontology Analysis

**Figure 6:**
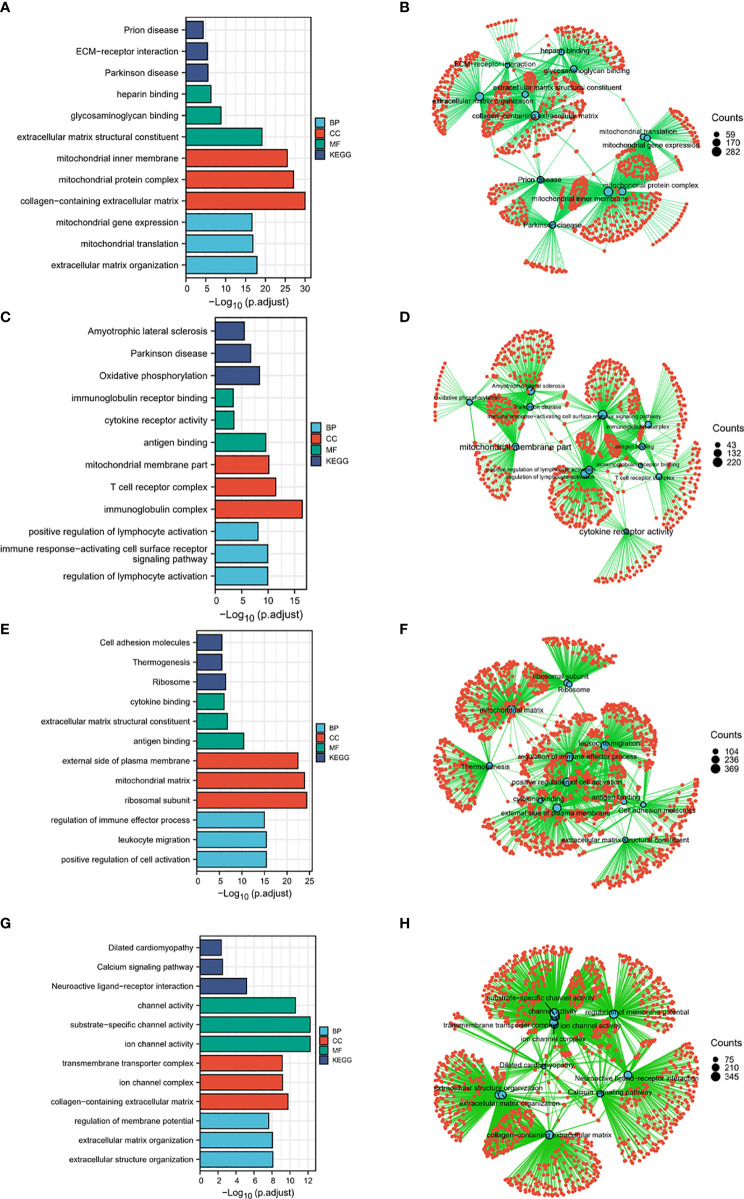
HSPA1A-, HSPA1B-, HSPA7-, and HSPA9-related signaling pathways identified by gene ontology. **(A)** and **(B)** HSPA1A-related signaling pathways. **(C)** and **(D)** HSPA1B-related signaling pathways. **(E)** and **(F)** HSPA7-related signaling pathways. **(G)** and **(H)** HSPA9-related signaling pathways.

To identify HSPA family members-related signaling pathways that were differently activated in colon cancer, we conducted Gene ontology (GO) Analysis (14). As illustrated in **Figure 6**, ECM-receptor interaction, heparin binding, glycosaminoglycan binding, extracellular matrix structural constituent, mitochondrial inner membrane, mitochondrial protein complex, collagen-containing extracellular matrix, mitochondrial translation, and extracellular matrix organization were potential signaling pathways regulated by HSPA1A (**Figures 6A, B**). These pathways indicated that HSPA1A may be involved in the regulation of mitochondrial and extracellular matrix-related processes. Oxidative phosphorylation, immunoglobulin receptor binding, cytokine receptor activity, antigen binding, mitochondrial membrane part, T cell receptor complex, immunoglobulin complex, positive regulation of lymphocyte activation, immune-response-activating cell surface receptor, and regulation of lymphocyte activation pathways were correlated with HSPA1B (**Figures 6C, D**). The result suggested that HSPA1B may take part in immune response processes. Pathways of cell adhesion molecules, thermogenesis, ribosome, cytokine binding, extracellular matrix structural constituent, antigen binding, external side of plasma membrane, mitochondrial matrix, ribosomal subunit, regulation of immune effector process, and leukocyte migration were related to HSPA7 (**Figures 6E, F**). HSPA7 may play roles in immune response and mitochondrial function in colon cancer. Calcium signaling pathway, neuroactive ligand–receptor interaction, channel activity, substrate-specific channel activity, ion channel activity, transmembrane transporter complex, ion channel complex, collagen-containing extracellular matrix, regulation of membrane potential, extracellular structure organization, and extracellular matrix organization were associated with HSPA9 (**Figures 6G, H**). HSPA9 may participate in the regulation of channel activity of colon cancer.

## Discussion

Although the systemic treatment of colon cancer has made some progress in recent years, such as the approval of PD-1 inhibitors, the prognosis of patients with advanced or recurrent disease is still poor (3). Discovery of new biomarkers with prognostic and therapeutic value is an urgent need.

Heat shock proteins (HSPs) are a group of proteins that function to reverse or inhibit denaturation or unfolding of cellular proteins in response to stress or high temperature. The HSPA family of HSPs, also known as the HSP70 family, encodes HSP70 proteins and plays important roles in the regulation of protein hemostasis by mediating correct protein folding. The HSP70 family is generally considered to be a stress-induced survival protein and be related to the enhancement of cell survival following a multitude of stresses (15). It has been reported that members of the HSPA family were related to tumor development and poor prognosis in some types of cancers, including colorectal cancer (16). However, the distinct roles of HSPA family members in colon cancer remain to be elucidated. Thus, in the current study, we analyzed the expression pattern and prognostic values of the distinct HSPA family members in colon cancer.

Results of our study showed that HSPA1B, HSPA4, HSPA5, HSPA6, HSPA8, HSPA9, HSPA13, and HSPA14 were upregulated, and those of HSPA1A, HSPA2, HSPA7, and HSPA12B were downregulated in colon cancer. Logistic regression showed that increased HSPA1A, HSPA1B, HSPA5, and HSPA6 were significantly related to the increased disease stage, and increased HSPA8 was related to the decreased stage of the disease. These results suggested that HSAPA1A may be associated with colon cancer progression rather than tumorigenesis. Kaplan–Meier survival analyses indicated that patients with increased HSPA1A, HSPA1B, and HSPA7 showed poor survival, while patients with increased HSPA9 showed favorable survival. Independent external datasets were used to verify the expression difference and the prognostic values of HSPA1A and HSPA9. The results were consistent with the results from the TCGA database, showing that HSPA1A was decreased and HSPA9 was increased in colon cancer. Increased HSPA1A and decreased HSPA9 were related to poor survival. DNA methylation is one of the most common epigenetic medications. Methylation of the promotor downregulates gene expression. To explore whether downregulation of HSPA1A and upregulation of HSPA9 in colon cancer were related to DNA methylation, we analyzed the methylation of HSPA1A and HSPA9 promoters. We found that HSPA1A promoter methylation was significantly increased and HSPA9 promoter methylation was significantly decreased in colon cancer. Downregulation of HSPA1A promoter methylation and upregulation of HSPA9 promoter methylation, which results in increased HSPA1A and decreased HSPA9 expression, were associated with poor survival. Colon cancer cell lines also showed increased expression of HSPA1B and HSPA9 and decreased expression of HSPA1A and HSPA7. The results were also consistent with the results from the TCGA database. However, it should be pointed out that we only analyzed the total methylation level and did not analyze the specific methylation sites of the promoter. In short, the above results indicated that increased HSPA1A was correlated with poor prognosis and increased HSPA9 was correlated with favorable prognosis in colon cancer.

Another study indicated that HSPA1A was decreased in colorectal cancer and high HSPA1A was related to poor survival (17). However, the results were not verified in an independent dataset. Our study verified the expression difference of HSPA1A in the protein level and the DNA methylation level as well as the correlation of HSPA1A with survival in independent datasets.

It has been indicated that HSPA1A plays an important role in tumor development. HSPA1A protects tumor cells from oxidative stress, inflammatory cytokines, hypoxia, and other stress (18). HSPA1A is involved in the promotion of tumor cell proliferation, metastasis, and invasion (19). Inhibition of HSPA1A promoted the apoptosis of colon cancer cells (20). Previous studies showed that HSPA1A was decreased in ovary cancer, and downregulation of HSPA1A was related to increased methylation. The increase in HSPA1A was correlated to poor prognosis of ovary cancer (21). The results were consistent with the current study.

It has been pointed out that HSPA1B was associated with risk and poor prognosis of lung cancer (22), and it was involved in the tumor growth of colorectal (23) and breast cancer (24). Our results, indicating that HSPA1B was related to poor survival of colon cancer, were consistent with previous studies. However, no independent dataset was founded to verify the results. The results need to be further validated.

HSPA9 is a heat-uninducible protein of the HSPA family, which plays critical roles in stress response, energy generation, neurodegenerative disease, and carcinogenesis (25). HSPA9 has been reported to be upregulated in liver cancer (26) and pancreatic cancer (27). It was reported to be related to metastasis and early recurrence in liver cancer (26). However, there was no report about HSPA9 expression and its prognostic value in colon cancer. Our study indicated that increased HSPA9 expression was associated with favorable survival in colon cancer. It should be noticed that HSPA9 was elevated in colon cancer tissues in comparison with nontumor tissues. The result gave a clue that HSPA9 may be related to the tumorigenesis rather than the progression of colon cancer.

It should be noticed that expression of HSPA7 was increased in colon cancer tissues in unpaired comparisons, but HSPA7 expression did not show statistical difference in paired comparisons. In the paired comparison, tumor samples and non-tumor samples were obtained from the same patient. The results from paired comparison were more persuasive than the results from nonpaired comparison. Moreover, although Cox regression showed that the upregulation of HSPA7 was related to poor survival in colon cancer, we did not find any independent dataset to verify the results. In addition, another database showed that HSPA7 displayed lower expression at the mRNA level but displayed no protein expression (https://platform.opentargets.org/target/ENSG00000225217). It is inconsistent with our research results. Thus, the expression difference of HSPA7 between colon cancer tissues and normal tissues and the relationship of HSPA7 with survival in colon cancer need further clinical and experimental verification.

In conclusion, the increased expression of HSPA1A1, HSPA1B, and HSPA7 was associated with poor prognosis, and HSPA9 was related to favorable prognosis for colon cancer. The current study is a bioinformatic study based on a public database, though the results were verified using an independent dataset; further *in vitro* and *in vivo* experiments are needed to confirm the result and explore the underlying mechanisms. Though the prognostic value of the HSPA family has been reported in some cancers, our research is the first one to demonstrate the significance of distinct HSPA family members in colon cancer and verify the result by several independent datasets in different expression levels.

### Public Database and Tools Used in the Current Study

1. The Cancer Genome Atlas (TCGA) database. Link: https://portal.gdc.cancer.gov/
2. Clinical Proteomic Tumor Analysis Consortium (CPTAC) database. Link: http://ualcan.path.uab.edu/analysis-prot.html
3. Gene Expression Omnibus (GEO) database. Link: www.ncbi.nlm.nih.gov/geo/
4. UALCAN. Link: http://ualcan.path.uab.edu/index.html
5. MethSurv. Link: https://biit.cs.ut.ee/methsurv/

## Data Availability Statement

The original contributions presented in the study are included in the article/**Supplementary Material**. Further inquiries can be directed to the corresponding authors.

## Author Contributions

YG, XZ, JL, MW, SH, and XP had full access to all the data in the study and take responsibility for the integrity of the data and the accuracy of the data analysis. YG, XZ, JL, MW, SH, and XP designed the study. Acquisition of data: YG, XZ, and JL. Analysis and interpretation of data: YG, XZ, JL, and MW. Statistical analysis: YG, XZ, JL, and MW. Drafting and revising of the manuscript: MW, SH, and XP. All authors contributed to the article and approved the submitted version.

## Funding

The study was supported by the National Science Foundation of Guangdong Province China (Grant No. 2020A1515110923).

## Conflict of Interest

The authors declare that the research was conducted in the absence of any commercial or financial relationships that could be construed as a potential conflict of interest.

## Publisher’s Note

All claims expressed in this article are solely those of the authors and do not necessarily represent those of their affiliated organizations, or those of the publisher, the editors and the reviewers. Any product that may be evaluated in this article, or claim that may be made by its manufacturer, is not guaranteed or endorsed by the publisher.

## Supplementary Material

Supplementary Figure 1 Correlation of HSPA family expression with clinical pathological characteristics in colon cancer. Correlation of HSPA1A, HSPA1B, HSPA2, HSPA4, HSPA5, HSPA6 and HSPA7 expression with **(A)** T stage, **(B)** N stage, **(C)** M stage, **(D)** pathologic stage, **(E)** age, **(F)** gender, **(G)** CEA level. (Wilcoxon rank sum test for T stage, N stage, M stage, age, gender and CEA level. Kruskal-Wallis test for pathologic stage. ns, non significant; **P* < 0.05; ***P* < 0.01; ****P* < 0.001).

Supplementary Figure 2 Correlation of HSPA family expression with clinical pathological characteristics in colon cancer. Correlation of HSPA8, HSPA9, HSPA12A, HSPA12B, HSPA13 and HSPA14 expression with **(A)** T stage, **(B)** N stage, **(C)** M stage, **(D)** pathologic stage, **(E)** age, **(F)** gender, **(G)** CEA level. (Wilcoxon rank sum test for T stage, N stage, M stage, age, gender and CEA level. Kruskal-Wallis test for pathologic stage. ns, non significant; **P *< 0.05; ***P* < 0.01; ****P* < 0.001).

Supplementary Figure 3 Survivals analysis of patients with different HSPA family member expression levels by Kaplan-Meier method. **(A)** Overall survival for HSPA2. **(B)** Overall survival for HSPA4. **(C)** Overall survival for HSPA5. **(D)** Overall survival for HSPA6. **(E)** Overall survival for HSPA8. **(F)** Overall survival for HSPA12A. **(G)** Overall survival for HSPA12B. **(H)** Overall survival for HSPA13. **(I)** Overall survival for HSPA14. **(J)** Progress free interval for HSPA2. **(K)** Progress free interval for HSPA4. **(L) **Progress free interval for HSPA5. **(M)** Progress free interval for HSPA6. **(N)** Progress free interval for HSPA8. **(O)** Progress free interval for HSPA12A. **(P)** Progress free interval for HSPA12B. **(Q)** Progress free interval for HSPA13. **(R)** Progress free interval for HSPA14.
