# Supplementary figures and images for: Upregulation of HSPA1A/HSPA1B/HSPA7 and Downregulation of HSPA9 Were Related to Poor Survival in Colon Cancer
Source: Front Oncol. 2021 Oct 26;11:749673. doi: 10.3389/fonc.2021.749673 (PMC8576338; doi:10.3389/fonc.2021.749673)

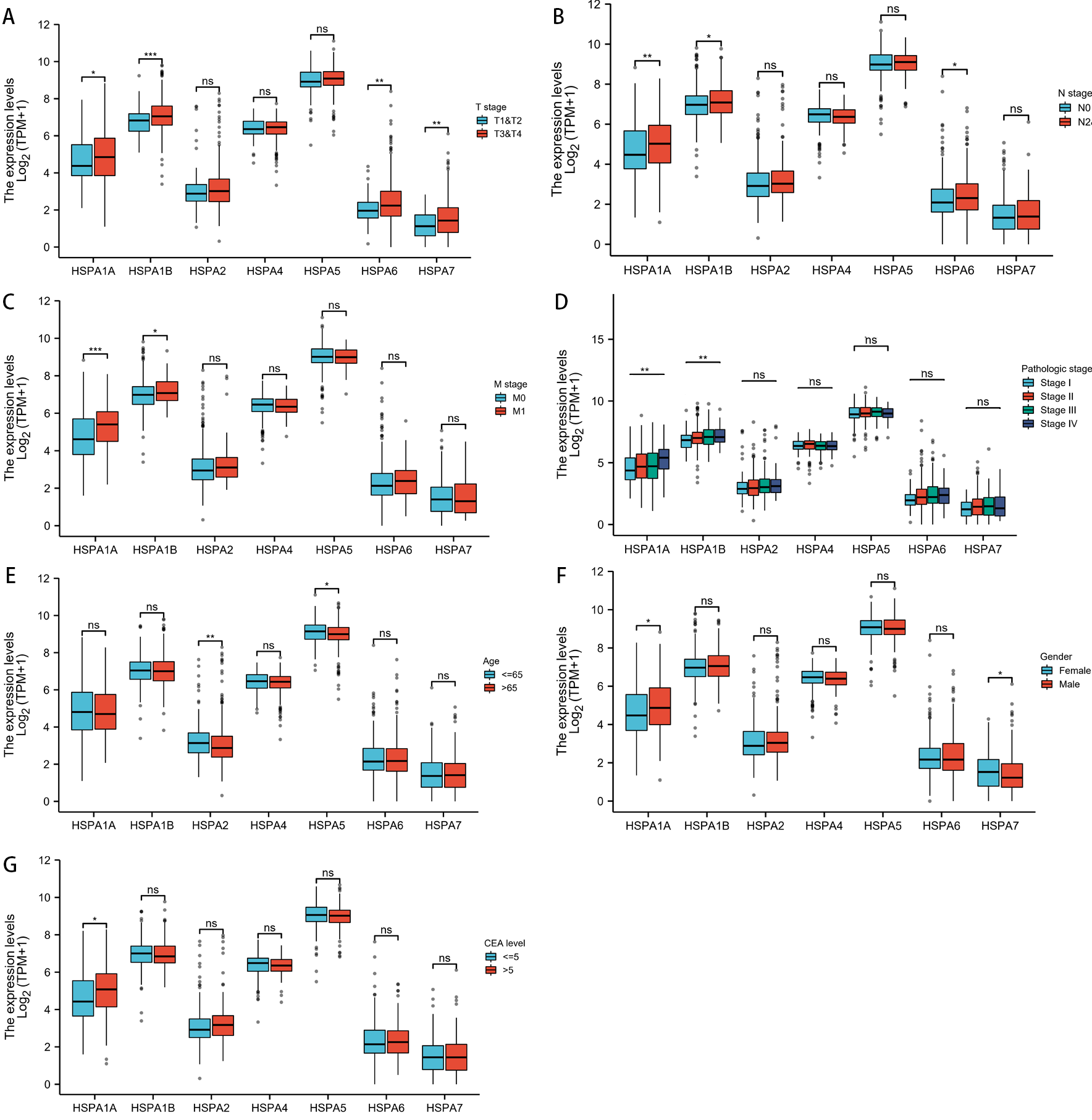

Supplement: Supplementary Figure 1 — Correlation of HSPA family expression with clinical pathological characteristics in colon cancer. Correlation of HSPA1A, HSPA1B, HSPA2, HSPA4, HSPA5, HSPA6 and HSPA7 expression with (A) T stage, (B) N stage, (C) M stage, (D) pathologic stage, (E) age, (F) gender, (G) CEA level. (Wilcoxon rank sum test for T stage, N stage, M stage, age, gender and CEA level. Kruskal-Wallis test for pathologic stage. ns, non significant; *P < 0.05; **P < 0.01; ***P < 0.001). [file Image_1.tif]

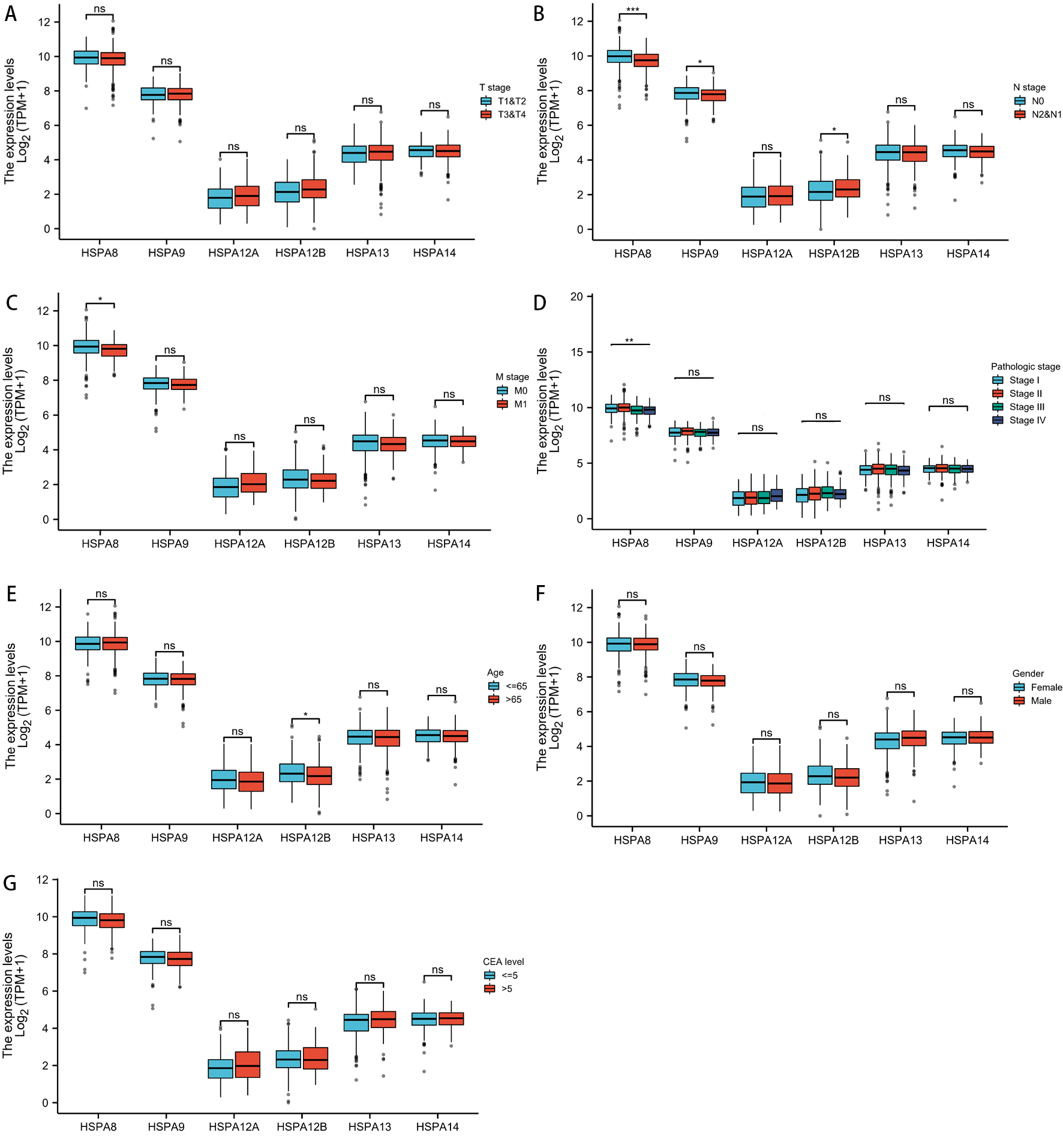

Supplement: Supplementary Figure 2 — Correlation of HSPA family expression with clinical pathological characteristics in colon cancer. Correlation of HSPA8, HSPA9, HSPA12A, HSPA12B, HSPA13 and HSPA14 expression with (A) T stage, (B) N stage, (C) M stage, (D) pathologic stage, (E) age, (F) gender, (G) CEA level. (Wilcoxon rank sum test for T stage, N stage, M stage, age, gender and CEA level. Kruskal-Wallis test for pathologic stage. ns, non significant; *P < 0.05; **P < 0.01; ***P < 0.001). [file Image_2.tif]

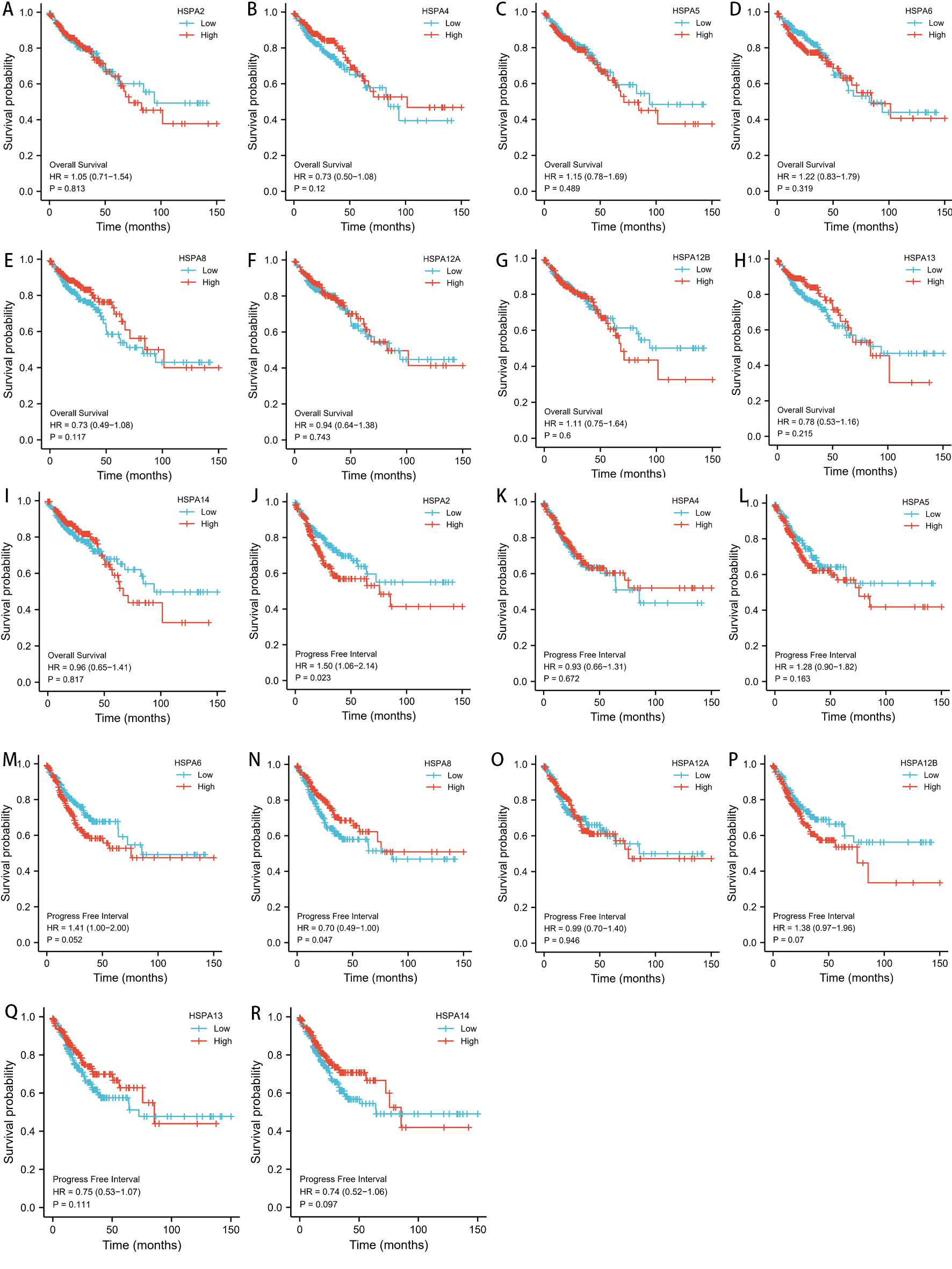

Supplement: Supplementary Figure 3 — Survivals analysis of patients with different HSPA family member expression levels by Kaplan-Meier method. (A) Overall survival for HSPA2. (B) Overall survival for HSPA4. (C) Overall survival for HSPA5. (D) Overall survival for HSPA6. (E) Overall survival for HSPA8. (F) Overall survival for HSPA12A. (G) Overall survival for HSPA12B. (H) Overall survival for HSPA13. (I) Overall survival for HSPA14. (J) Progress free interval for HSPA2. (K) Progress free interval for HSPA4. (L) Progress free interval for HSPA5. (M) Progress free interval for HSPA6. (N) Progress free interval for HSPA8. (O) Progress free interval for HSPA12A. (P) Progress free interval for HSPA12B. (Q) Progress free interval for HSPA13. (R) Progress free interval for HSPA14. [file Image_3.tif]
