# Supplementary material for: Upregulation of HSPA1A/HSPA1B/HSPA7 and Downregulation of HSPA9 Were Related to Poor Survival in Colon Cancer
Source: Front Oncol. 2021 Oct 26;11:749673. doi: 10.3389/fonc.2021.749673 (PMC8576338; doi:10.3389/fonc.2021.749673)
Supplement: Supplementary file 4 [file Table_1.docx]

**Table S1 Patient characteristics**

| Characteristics | | N | Percentage (%) |
| --- | --- | --- | --- |
| Age | >65y | 284 | 59.17 |
|  | <=65y | 194 | 40.42 |
|  | unknown | 2 | 0.42 |
| Gender | Female | 226 | 47.08 |
|  | Male | 252 | 52.50 |
|  | unknown | 2 | 0.42 |
| T stage | 1 | 11 | 2.29 |
|  | 2 | 83 | 17.29 |
|  | 3 | 323 | 67.29 |
|  | 4 | 60 | 12.50 |
|  | unknown | 3 | 0.63 |
| N stage | 0 | 284 | 59.17 |
|  | 1 | 108 | 22.50 |
|  | 2 | 86 | 17.92 |
|  | unknown | 2 | 0.42 |
| M stage | 0 | 349 | 72.71 |
|  | 1 | 66 | 13.75 |
|  | unknown | 65 | 13.54 |
| Pathologic stage | I | 81 | 16.88 |
|  | II | 187 | 38.96 |
|  | III | 133 | 27.71 |
|  | IV | 66 | 13.75 |
|  | unknown | 13 | 2.71 |
| CEA (ng/ml) | <=5 | 195 | 40.63 |
|  | >5 | 107 | 22.29 |
|  | unknown | 178 | 37.08 |
| Total |  | 480 | 100.00 |
